# Assessing self-stigma levels and associated factors among substance use disorder patients at two selected psychiatric hospitals in Egypt: a cross-sectional study

**DOI:** 10.1186/s12888-023-05093-0

**Published:** 2023-08-15

**Authors:** Ibrahem Hamdey Rashed Elkalla, Abdel-Hady El-Gilany, Mohamed Baklola, Mohamed Terra, Mostafa Aboeldahab, Samir El Sayed, Mohammed ElWasify

**Affiliations:** 1 Psychiatry Department, Faculty of Medicine, Mansoura University, 60El-Gomhoria Street, Mansoura, 35516 Egypt; 2 Public Health and Community Medicine Department, Faculty of Medicine, Mansoura University, 60El-Gomhoria Street, Mansoura, 35516 Egypt; 3 Faculty of Medicine, Mansoura University, 60El-Gomhoria Street, Mansoura, 35516 Egypt; 4 Port-Said Mental Health and Addiction Treatment Hospital, Port-Said, Egypt

**Keywords:** Substance use, Perceived stigma, Clinical history parameters, Treatment access, Education, Awareness

## Abstract

**Background:**

Substance use disorder is a growing problem worldwide, and the stigma associated with it remains a significant barrier to treatment and recovery. This study aimed to assess the perceived stigma among individuals with substance use disorders and its correlation with their socio-demographic characteristics and clinical history Parameters.

**Methods:**

A cross-sectional study was conducted among 552 patients with substance use disorders admitted to the outpatient clinics of Mansoura University Hospital, Addiction Treatment Unit of the Psychiatry Department, and Port Said Mental Hospital, Addiction Department. Participants completed a self-administered questionnaire, which included demographic information, clinical history parameters, and the Perceived Stigma of Substance Abuse Scale (PSAS).

**Results:**

The study found that almost half of the participants were aged 29 or younger, married, and had a median stigma score of 20. The vast majority of participants were male, had no previous legal problems, and had a median stigma score of 19. The most common type of substance used was opioids, and more than half of the participants were still using drugs. The highest mean stigma scores were for the items “Most people think less of a person who has been in treatment for substance use” and “Most employers will pass over the application of someone who has been treated for substance use in favor of another applicant.“ The perceived stigma score was significantly correlated with the severity of use but not with age or duration of use.

**Conclusion:**

Our study investigates self-stigma in substance use disorder (SUD), revealing its variance across demographics and clinical groups. We found that self-stigma correlates with use severity and possibly decreases with abstinence. Notably, societal bias contributes significantly to self-stigma, necessitating societal interventions. The impact of self-stigma on patient well-being highlights the need for personalized treatments and stigma reduction strategies.

## Background

The world is currently facing a growing issue of substance use disorder, which has become a significant public health concern [1]. Substance use disorder is a complex disease that affects individuals, families, and society as a whole. It is characterized by repeated use of substances such as alcohol, tobacco, and other drugs, despite the harmful consequences that can result from such use [2, 3].

Individuals with substance use disorder face various challenges, including the chronic and complicated course of the disease, which can have significant social and economic impacts, even after recovery [4]. One of the significant challenges is stigma, especially self-stigma, which can be an additional burden on the recovery process of patients with substance use disorder [5]. Stigma can lead to delays in seeking treatment, diminished self-worth and self-efficacy, and lower quality of life. The fear of community stigma can also hinder individuals from seeking treatment [5].

Individuals with substance use disorders often experience different forms of stigma. According to Corrigan and colleagues [6], three categories of stigma have been identified within this population: [1] public stigma, which refers to prejudice and discrimination from the general population that negatively affects an individual; [2] self-stigma, which is the harm that occurs when a person internalizes this prejudice; and [3] structural stigma, which encompasses the policies of private and governmental institutions that intentionally restrict the opportunities available to individuals with mental illness. Structural stigma can also refer to the unintended consequences of policies adopted by major institutions, which inadvertently hinder the options of people with mental illness [6].

Self-stigma, or the internalization of social invalidation, is an integral component in the context of substance use disorder [7]. It manifests through self-criticism and feelings of shame, which can further foster the perception of being flawed, disrespected, and rejected. The resultant shame acts as a barrier to social engagement, leading to interpersonal dissociation and hindering problem-solving abilities in interpersonal situations [8].

One salient mechanism underpinning this stigma is psychological inflexibility. This pertains to an individual’s inability to fully acknowledge their present experiences and engage in behaviors that align with their chosen values [9]. This psychological dynamic can manifest as self-stigma, obstructing individuals from living a life congruent with their values. For instance, the fear of mistrust due to their disease history can deter individuals from seeking treatment or engaging in intimate relationships [8].

Furthermore, this internalized stigma acts as a significant obstacle to recovery. It can dissuade individuals from actively participating in recovery communities, thus impeding their pathway toward healing and reintegration into society [10].

Although the evidence that self-stigma hinders treatment seeking and affects outcomes for patients with substance use disorder is well established, there are no systematic reviews assessing the actual prevalence of its impact. Previous studies in the field of psychological stigma have illustrated different perspectives on the impact of stigma. For example, patients with substance use disorders are often accused of being responsible for their condition and its consequences, leading to more negative emotions and making them more susceptible to social discrimination [11].

Recovery from substance use disorder is a sustained process that includes all family members, rather than just the patient [12]. Recovery from the damage caused by the disease and the consequence of its stigma requires believing that recovery is a continuing process of achieving values and fighting psychological and social factors of relapse [13].

The importance of the motivational process and its impact on the treatment of substance use disorder patients are significant, as it requires patients to be actively engaged in cognitive and emotional techniques to progress through the initial phases and to proceed and sustain the change. There are concerns about how the process of consistent behavioral change involves resolving the inner feeling of self-shame that may emerge at any stage in the recovery process [14]. Thus, continuous evaluation of perceived stigma is important at every stage of the recovery process [15].

Therefore, the objective is to measure the level of stigma among substance use disorder patients using the validated Arabic version of the Perceived Stigma of Substance Abuse Scale (PSAS) and identify the factors that are associated with higher levels of perceived stigma among this population, which will help in understanding the underlying causes and inform the development of more effective interventions.

## Methods

### Study design, setting, and study period

A descriptive, cross-sectional study with an analytic component was conducted from December 2020 to December 2022. This research was carried out in the outpatient clinics of the Addiction Treatment Unit of the Psychiatry Department at Mansoura University Hospital, as well as the Addiction Department at Port Said Mental Hospital.

### Inclusion and exclusion criteria

This study included individuals who are 18 years of age or older, from both genders. The participants needed to have a history of regular substance use for a minimum of one year and meet the diagnostic criteria for substance use disorder according to the Structured Clinical Interview for DSM-IV Axis I Disorders (SCID-I) [16].

Urine samples were collected from participants at the time of recruitment for a urine drug screen test using Abon Biopharm Multi-Drug Screen kits. The screen targeted substances such as opioids, tramadol, cannabis, amphetamines, and benzodiazepines. This test aimed to verify the continued use in participants and confirm abstinence in those undergoing rehabilitation. This was to ensure that even individuals who were abstinent at the time of recruitment but had a history of substance use could be included.

We made a deliberate effort to include participants from various stages of their treatment journey, from those in the detoxification stage to others in the rehabilitation stage. As for the exclusion criteria, participants diagnosed with any other Axis I Disorders (according to SCID-I) beyond substance use disorder were not included in this study. Moreover, patients who were unwilling or unable to fully participate in the study or share the necessary information were also excluded.

These criteria were designed to ensure a diverse participant pool that had a history of substance use. This approach aimed to capture the changing perceptions around stigma associated with substance use at different stages of treatment, thereby providing a comprehensive understanding of the transformation occurring during the recovery process.

### Sample size

The sample size was calculated using Medcalc 15.8. The primary outcome of interest was the mean stigma score. A pilot study on 30 subjects revealed that the mean stigma score (SD) is 20.3 (5.1), with an alpha error of 5%, study power of 90%, and 5% precision, then the minimum required sample size is 265. This was multiplied by a design effect of 2, then the final minimum required sample size was 530. We recruited 552.

### Sampling method

Consecutive sampling was used to recruit patients from the attendants of the hospitals mentioned above. Before this, a jury and pilot study were conducted to assess the reliability of the translated tool and to evaluate its content validity and internal consistency. Patients of the pilot study were not included in the full-scale study.

### Study tools

The current study employed a self-administered questionnaire as a tool for data collection on various socio-demographic and clinical variables. Socio-demographic variables included age, sex, residence, education, occupation, and marital status. Clinical variables consisted of the type of substance the patient was dependent on, the duration of substance use disorder, how frequently the patient was taking the substance, the patient’s current state of dependence or abstinence, the number of attempts made by the patient to be abstinent, previous admission to an addiction unit, the longest period of abstinence, attempts to be abstinent outside the addiction unit, legal problems, psychiatric symptoms, and any other medical diseases.

The diagnosis of substance use disorder criteria and exclusion of other Axis I disorders were based on the gold standard Structured Clinical Interview for DSM-IV Axis I Disorders (SCID-I), a psychiatric disorder diagnosis tool preferred by most psychiatrists in Egypt [15, 17]. Participants also completed the Severity of Dependence Scale (SDS), a brief and user-friendly tool that assesses the level of dependence experienced by individuals using different types of drugs. The SDS includes five items, each focusing on a specific psychological aspect of dependence, such as preoccupation and anxiety related to drug use [18–20].

To measure perceived stigma in substance-use disorder patients, the Perceived Stigma of Substance Abuse Scale (PSAS) was utilized. The PSAS is an eight-item scale with good face and construct validity and adequate levels of internal consistency [21].

The scale was originally developed in English by Luoma et al. [21], and assesses perceived stigma along a 4-point Likert-type scale ranging from strongly disagree to strongly agree. The scale provides a single total score, with higher scores indicating greater perceived stigma. Reversed scored items include 1, 2, 3, 4, 6, and 8 [21].

### Translation and validation of PSAS

The process of translation and cultural adaptation of the Perceived Stigma of Substance Abuse Scale (PSAS) followed international guidelines for cross-cultural adaptation of health questionnaires [22].

This involved a step-by-step approach, which included forward translation, synthesis of the translated versions, back translation, an expert committee, and a test of the pre-final version. The conceptual framework of the scale was established by obtaining the opinions of a jury of 10 experts in psychiatry, comprising three professors, two assistant professors, four consultants, and one assistant lecturer.

**Fig. 1:**
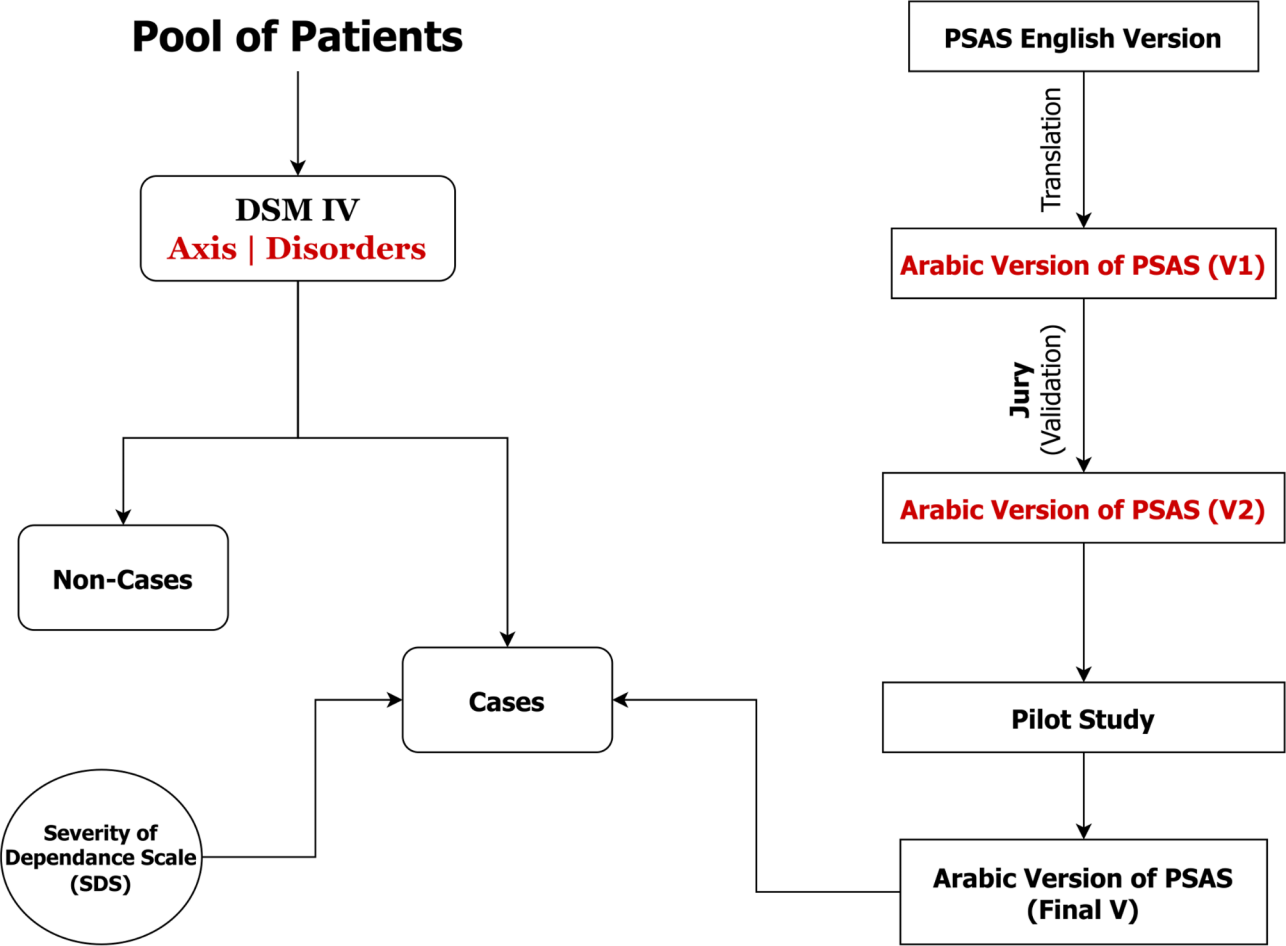
From Translation to Application: A Visual Guide to Methodology for Assessing Stigma among Substance abusers

The Arabic version of the PSAS scale was then evaluated for clarity and relevance of content, with the content validity index (CVI) calculated at the item level (I-CVI) and expert level (E-CVI) [23]. The I-CVI ranged from 0.95 to 1 for clarity and from 0.91 to 1 for relevance, while the E-CVI ranged from 0.83 to 1 for both clarity and relevance, details are shown in Table 1. Furthermore, from the pilot study, internal consistency was examined using Cronbach’s α reliability coefficient, which was calculated to be 0.77, indicating moderate to good reliability. An overview of the entire study process is shown in Fig. 1.

**Table 1:** Content validity indices of the Arabic version of perceived stigma of substance abuse assessment scale (PSAS)

Item	I-CVI for relevance	I-CVI for clarity	Expert	E-CVI for relevance	E-CVI for clearance
**1**	1	1	**1**	1	1
**2**	1	1	**2**	1	1
**3**	1	0.91	**3**	1	1
**4**	0.96	1	**4**	1	1
**5**	0.92	0.96	**5**	0.83	0.83
**6**	0.96	0.96	**6**	0.96	0.96
**7**	1	1	**7**	1	1
**8**	1	0.92	**8**	1	1
			**9**	0.83	0.83
			**10**	1	1
** S-CVI/Ave**	**0.96**	**0.96**			
** S-CVI/UA**	**0.87**	**0.87**			

IQR=Interquartile range (Q3-Q1). A & B significant difference between the corresponding groups by post-hoc analysis. * Kruskal-Wallis test

### Statistical analysis

Data were analyzed using the Statistical Package for Social Science Program (SPSS 25 for Windows). Categorical variables were presented as numbers and percentages. Numerical variables were presented as mean (SD) as well as median (inter-quartile range). Mann-Whitney and Kruskal-Wallis tests were used for comparison of the stigma score between categories, as appropriate. The Spearman correlation coefficient was used to measure the correlation between numerical variables. P ≤ 0.05 was considered statistically significant.

## Results

### Participant’s demographic characteristics and clinical history Parameters, variations of total stigma score according to these parameters

Of the 552 participants included in this study, almost half of them were at the age of 29 or younger and married with median stigma scores of 20, and 19 (IQR = 5, 6), respectively. The age of the study sample ranged from 18 to 61 years with a mean of 29.9 (± 8). The vast majority of the participants were males, with no previous legal problems with a percent of 88.5%, 82.4%, and a median stigma score of 19 (IQR = 6). Nearly one-third of the participants were at an educational level of less than secondary school and tried to be abstinent three times or more with a median stigma score of 19 (IQR = 5, 5.5), respectively.

Regarding the participants, a relatively large proportion of the participants are still users of drugs with a percentage of 57, and a median stigma score of 20 (IQR = 6). Only a small portion of the participants had psychiatric symptoms (25%) with a median stigma score of 19 (IQR = 5). There was a significant difference regarding the Hospital, sex, education, occupation, residence, if he was still actively dependent, number of trials to be abstinent, trial to be abstinent outside the addiction unit, period of the longest time to be abstinent, legal problems, and severity of dependence in terms of the stigma score. More details regarding the characteristics of the participants are shown in Table 2.

**Table 2:** Sociodemographic and clinical history Parameters, variations of total stigma score according to these parameters

Variables		Total N (%)	Stigma Score Median (IQR)	P-value
Overall	552		19 (5)	
**Location of study**	Mansoura University Hospital	328 (59.4)	19 (5)	**≤ 0.001**
	Port said Mental Hospital	224 (40.6)	20 (7)	
**Sociodemographic data**				
**Age (years)**	< 30	278 (50.3)	20 (5)	0.2
	30 or more	274 (49.7)	19 (5)	
**Sex**	Male	489 (88.5)	19 (6)	**0.002**
	Female	63 (11.5)	20 (6)	
**Education**	< 2ry	194 (35.2)	19 (5)	0.02*
	2ry	222 (40.2)	19 (7)^A^	
	> 2ry	136 (24.6)	20 (5.75) ^B^	
**Occupation**	Non-working/housewives	135 (24.5)	19 (7)	0.1*
	Farmer/manual workers	261 (47.2)	19 (5)	
	Professional/semi-professional	156 (28.3)	20 (7.75)	
**Are you Currently Married**	No	275 (49.8)	19 (6)	0.7
	Yes	277 (50.2)	19 (6)	
**Residence**	Urban	315 (57)	19 (6)	**≤ 0.001**
	Rural	237 (43)	20 (6)	
**Clinical Data**				
**Do you still actively use substances**	Users	315 (57)	20 (6)	**0.001**
	Abstinent	237 (43)	19 (6)	
**Trial to be abstinent**	No	112 (20.5)	20 (5.75) ^A^	0.01*
	1	151 (27.5)	19 (6)	
	2	120 (21.7)	19 (6)^B^	
	3 or more	169 (30.3)	19 (5.5)	
**Previous admission to hospital**	No	361 (65.3)	19 (6)	0.2
	Yes	191 (35.7)	19 (6)	
**Were you tried to be abstinent outside the addiction unit**	No	202 (36.5)	20 (6)	**0.03**
	Yes	350 (64.5)	19 (6)	
**longest time to be abstinent (in years)**	No	103 (18.6)	20 (7)^A^	0.008*
	1	232 (42)	19 (5.75)	
	2 or more	217 (39.4)	19 (7)^B^	
**Do you have Legal problems**	No	455 (82.4)	19 (6)	**≤ 0.001**
	Yes	97 (17.6)	21 (5)	
**Do you have Psychiatric symptoms**	No	414 (75)	19 (5)	0.7
	Yes	138 (25)	19 (5)	
**Do you have other medical diseases**	No	427 (77.3)	19 (6)	0.2
	Yes	125 (22.7)	19 (6)	
**Number of substances**:	Single	477 (86.5)	19 (6)	0.07
	Poly	75 (13.5)	20 (7)	
**Type of Substance patient is dependent on**	Opiates	379 (68.6)	19 (5)^A^	0.01*
	Cannabinoids	86 (15.5)	20 (7.25) ^B^	
	Alcohol/Benzodiazepines	12 (2.4)	21 (6.5)	
	Poly substance	75 (13.5)	20 (7)	
**Substance dependency disorder duration (years)**	< 10	257 (46.5)	19 (5)	0.76
	10 or more	295 (53.5)	19 (6)	
**Severity of dependence scale (SDS)**	< 10	207 (37.5)	17 (3)	**≤ 0.001**
	10 or more	345 (63.5)	21 (4)	

*Note*. SA = strongly agree, A = agree, D = disagree, SD = strongly disagree. IQR = Interquartile range (Q3-Q1)

Study results demonstrated a significant difference between specific groups. The self-stigma levels were significantly higher among opiate-dependent patients compared to those with cannabinoid dependency. However, there were no significant differences in self-stigma levels between patients with opiate and alcohol dependencies, nor between patients with alcohol, polysubstance, and cannabinoid dependencies.

### Perceived stigma of substance abuse scale

“Most people think less of a person who has been in treatment for substance use” and “Most employers will pass over the application of someone who has been treated for substance use in favor of another applicant” were the items with the highest mean score of stigma. More details of all other items of the scale are shown in Table 3.

**Table 3:** Descriptive statistics of perceived stigma of substance abuse assessment scale (PSAS)

Item	SA N (%)	A N (%)	D N (%)	SD N (%)	Mean ± SD	Median (IQR)
1. Most people would willingly accept someone who has been treated for substance use as a close friend.	122 (22.1)	243 (44.0)	119 (21.6)	68 (12.3)	2.24 ± 0.9	2(1)
2. Most people believe that someone who has been treated for substance use is just as trustworthy as the average citizen.	12(23.2)	241(43.7)	126(22.8)	57(10.3)	2.2 ± 0.9	2(1)
3. Most people would accept someone who has been treated for substance use as a teacher of young children in a public school.	94 (17.0)	194 (35.1)	169 (30.6)	95 (17.2)	2.5 ± 1.0	2(1)
4. Most people would hire someone who has been treated for substance use to take care of their children	87 (15.8)	202 (36.6)	164 (29.7)	99 (17.9)	2.5 ± 1.0	2(1)
5. Most people think less of a person who has been in treatment for substance use.	92 (16.7)	134 (24.3)	229 (41.5)	97 (17.6)	2.6 ± 1.0	2(1)
6. Most employers will hire someone who has been treated for substance use if he or she is qualified for the job.	99 (17.9)	279 (50.5)	126 (22.8)	48 (8.7)	2.2 ± 0.8	2(1)
7. Most employers will pass over the application of someone who has been treated for substance use in favor of another applicant.	64 (11.6)	144 (26.1)	236 (42.8)	108 (19.6)	2.7 ± 0.9	2(1)
8. Most people would be willing to date someone who has been treated for substance use.	133 (24.1)	213 (38.6)	141 (25.5)	65 (11.8)	2.3 ± 1.0	2(1)
**Total score**					19.2 ± 4.9	19(6)

### Correlation between perceived stigma and some participants’ characteristics

The correlation analysis demonstrated a significant moderate positive correlation between the perceived stigma score and the severity of the dependence (r = 0.69) while showing a weak negative correlation with the period of suspension (r= -0.12). There was no significant correlation regarding age, the number of trials to be abstinent, and the duration of dependence as shown in Table 4.

**Table 4:** Correlation between total stigma score and some parameters

	Stigma sore	
	*r*	*P-value*
**Age**	-0.02	0.6
**Number of trials to be abstinent**	-0.03	0.5
**Longest period to be abstinent**	-0.12	**0.005**
**Substance dependency disorder duration**	0.01	0.9
**Severity of dependence scale (SDS)**	0.69	**≤ 0.001**

## Discussion

Addiction is a medical condition that is unfortunately stigmatized, causing individuals to avoid seeking treatment. Despite increasing worldwide attention, the addiction-related stigma persists [24]. Such stigma has significant negative effects on individuals suffering from substance use disorders. In the context of addiction treatment, the stigma surrounding drug dependence is a significant obstacle. Stigma, whether experienced externally or internalized, can impede a person’s decision to pursue and sustain treatment programs [25, 26].

Stigma is divided into three categories: public stigma, self-stigma, and structural stigma [27]. Public stigma refers to the prejudice and discrimination experienced by individuals from the general population, which negatively impacts their lives. Self-stigma occurs when individuals internalize the stigma and discrimination directed toward them, causing harm to their mental health and self-esteem [7]. Structural stigma is exhibited through the policies of private and governmental institutions that intentionally limit the opportunities and resources available to individuals with addiction. Although these policies are intended to organize and protect society, they may unintentionally restrict the opportunities of individuals with addiction [27].

Our study included a sample of patients who were currently using drugs, as well as those who were at various stages of recovery. The overall mean stigma score was found to be 19.2, with a median of 19. These findings were similar to those of a previous Italian study that examined perceived stigma related to drug use among students and healthcare workers in Italy and Belgium. The study utilized the PSAS questionnaire and found that the mean perceived stigma score was 23.68 among Italians and 20.26 among Belgians, respectively [28].

Another study was conducted to compare the perceived stigma between substance use disorder and other mental disorders [29]. The findings revealed that addiction was associated with higher levels of perceived stigma compared to other mental disorders. Individuals with substance use disorders were perceived as more dangerous, more responsible for their condition, and less likely to receive help than those with other psychiatric conditions [29].

Numerous factors can affect the perception of stigma among individuals with substance use disorders. The current study found no statistical difference in the perception of stigma between different age groups. A systematic review and meta-analysis also found that age was not associated with an increased self-reported stigma in individuals with mental disorders, including substance use disorders [30].

However, the public stigma towards individuals with substance use disorders was found to increase with age [30]. This may be due to younger individuals being perceived as less responsible for their drug use compared to older individuals who may be discriminated against due to the belief that they should have known better [30]. Additionally, older individuals may face more societal pressure to overcome their addiction, as they are seen as having had more time to address the issue. Conversely, younger individuals may be viewed as more susceptible to peer pressure and less experienced in managing their addiction [30].

According to the current study, females report experiencing more perceived stigma compared to males. This is consistent with other research findings that suggest women who use cannabis and amphetamines experience more stigma and may be more likely to face avoidance and coercion than men [31]. It also found a statistically significant difference in perceived stigma between different substances of abuse. Substances considered stronger, such as heroin, were associated with more perceived stigma compared to cannabis [31]. This is likely due to the perception that individuals who use stronger substances are more reckless, face a higher risk of severe negative consequences, and have greater difficulty in stopping their addiction.

The findings of our study illuminate important distinctions in the experience of self-stigma among patients with different substance use disorders. A marked difference was observed between opiate-dependent patients and those with cannabinoid dependence. Self-stigma, the internalized sense of shame or embarrassment associated with one’s behavior or condition, was significantly more pronounced in the opiate-dependent group. The disparity in self-stigma levels between these two groups suggests unique psychological and social factors at play in the experience of opiate dependence versus cannabinoid dependence.

Our study’s findings align well with previously conducted study among Mental Health Professionals and Medical Students in Egypt [32]. It was observed in this previous study that marijuana had the least stigma among the substances they examined, while heroin carried the most, particularly in terms of social distance and the perception of danger associated with its use [32]. Indeed, there has been a growing acceptance of marijuana usage in society. Recent data indicate that the levels of perceived risk and disapproval of marijuana use among adults and adolescents have been on a steady decline [33, 34].

Opiates are generally viewed as more harmful, and their use is more stigmatizing, compared to cannabinoids. This societal bias may be mirrored in the higher self-stigma reported among opiate-dependent patients [35]. However, it is worth noting that no significant difference in self-stigma was identified between opiate-dependent and alcohol-dependent patients. This could be indicative of a similar level of stigma associated with these two types of substance dependence, possibly due to their severe health consequences and the social perceptions surrounding their use [35]. Similarly, there was no discernable difference in self-stigma levels among patients with alcohol, polysubstance, and cannabinoid dependencies. This absence of significant variation could suggest that the shared experience of dependence may engender comparable levels of self-stigma across these groups, irrespective of the specific substances involved [36].

The current study did not find a statistically significant difference in stigma scores related to patients who have psychiatric symptoms and other medical diseases. This may be due to a lack of awareness of mental symptoms and other medical diseases among the study sample. When analyzing the socio-demographics of the sample, it was found that patients from rural areas expressed more stigma than those from urban areas.

In addition, level of education was found to affect the presence of self-stigma, with higher-educated patients experiencing more self-stigma compared to those with lower levels of education in our sample group. This finding is consistent with a study that found individuals with higher educational levels reported higher levels of self-stigma [37].

In terms of occupational status, professional workers have higher stigma scores, but this difference is not statistically significant when compared to other groups. This may be interpreted as a sense of self-shame and isolation from their peers who share the same education and occupational status. This is supported by another study that found a positive correlation between loneliness and perceived stigmatization [38].

In our study, we did not find any statistically significant difference in self-stigma related to marital status, which is consistent with a previous study that also found no influence of marriage on self-stigma [39].

Regarding the treatment and rehabilitation process, our results showed that self-stigma was higher among active substance users and decreased among those in remission. Furthermore, individuals who had been in remission for longer periods reported lower levels of self-stigma. Interestingly, individuals who participated in outpatient programs reported lower levels of self-stigma than those who were admitted to inpatient programs. Additionally, self-stigma was lower among individuals who had attempted to quit substance use multiple times. The duration of addiction did not show a significant difference in terms of self-stigma, but the severity of dependence was found to increase the perception of self-stigma in our study.

All of this may be explained by the level of familiarity that individuals with substance use disorders have with their condition [40]. Those who engage in rehabilitation programs perceive their condition as less dangerous and express less fear, avoidance, and coercion, and are more willing to seek help.

These findings support the idea that contact with rehabilitation programs and familiarity with addiction as a disease rather than a shameful fault can improve attitudes and understanding toward individuals in the therapeutic community and towards oneself, ultimately decreasing self-stigma [41]. Further research is needed to understand the impact of the therapeutic community’s nature and quality on reducing self-stigma.

The presence of legal problems and/or imprisonment has been shown to have a statistically significant effect on self-stigma in individuals with substance use disorders. This is consistent with previous studies that have demonstrated an increase in self-stigma among individuals whose addiction problems intersect with the criminal justice system, which can worsen their mental and physical health and increase the likelihood of relapse after release [42]. In response to this burden, modern laws are aiming to decriminalize drug abuse problems and treat them as medical issues in order to reduce the stigma around drug dependence [43].

### Strength of the study

Translation and validation of PSAS: The Perceived Stigma of Substance Abuse Scale (PSAS) and recruitment of a large number of substance use disorder patients from two major hospitals in Egypt.

### Limitations

While our study provides useful insights into substance use patterns in our sample, it is important to acknowledge its limitations.

First, the cross-sectional design allows us to observe associations between variables at a single point in time, which restricts our ability to draw conclusions about causality or monitor changes over time.

Second, we collected data only from two hospitals in Egypt, a fact that potentially limits the generalizability of our findings to other settings or wider populations. This underlines the need for similar research in diverse contexts for broader representation.

Perhaps most significantly, our study utilized self-administered questionnaires for data collection. While this method was chosen due to its feasibility in our study context, it may introduce elements of response bias or social desirability bias, especially when assessing sensitive topics such as substance use. The potential for underreporting or overreporting in this context is a limitation. Future studies might benefit from incorporating more objective, clinician-rated measures like the Addiction Severity Index, a point also suggested by the peer review.

Lastly, we also acknowledge the limited representation of female participants in our study, which could affect the generalizability of our findings across genders. Future research must ensure balanced gender participation.

Recognizing these limitations is essential for the proper interpretation of our study and underscores the value of continued research in this field.

## Conclusion

This study explored the presence and complexity of self-stigma in individuals with substance use disorder (SUD), revealing significant variations across demographic and clinical subsets. Greater self-stigma was found in females, active substance users, and those with opiate dependence.

Self-stigma’s association with dependency severity and its possible reduction with sustained substance abstinence point to its therapeutic significance. Societal bias, particularly in employment contexts, was found to be a notable contributor to self-stigma, highlighting the need for societal-level interventions. The impact of self-stigma on patient well-being, treatment adherence, and quality of life stresses the necessity for tailored therapeutic approaches and stigma reduction strategies.

These findings underpin future research into the mechanisms underlying these relationships, critical for effective SDD management and stigma mitigation.

## Data Availability

The datasets used during the current study are available from the corresponding author upon reasonable request.

## References

[1] Lo TW, Yeung JWK, Tam CHL. Substance Abuse and Public Health: A Multilevel Perspective and Multiple Responses. Int J Environ Res Public Health [Internet]. 2020 Apr 1 [cited 2023 Jun 21];17(7). Available from: https://www.ncbi.nlm.nih.gov/pmc/articles/PMC7177685/. 10.3390/ijerph17072610 PMC7177685 32290248

[2] Sadock BJ, Sadock VA, Ruiz P, Kaplan. & Sadock’s comprehensive textbook of psychiatry. [cited 2023 Jun 21]; Available from: https://www.wolterskluwer.com/en/solutions/ovid/kaplan--sadocks-comprehensive-textbook-of-psychiatry-761.

[3] Forman RF, Svikis D, Montoya ID, Blaine J. Selection of a substance use disorder diagnostic instrument by the National Drug Abuse Treatment Clinical Trials Network. J Subst Abuse Treat [Internet]. 2004 [cited 2023 Jun 21];27(1):1. Available from: https://pubmed.ncbi.nlm.nih.gov/15223087/. 10.1016/j.jsat.2004.03.012 PMC2668155 15223087

[4] Daley DC. Family and social aspects of substance use disorders and treatment. J Food Drug Anal [Internet]. 2013 [cited 2023 Jun 21];21(4):S73. Available from: https://www.ncbi.nlm.nih.gov/pmc/articles/PMC4158844/. 10.1016/j.jfda.2013.09.038 PMC4158844 25214748

[5] Luoma JB, Kohlenberg BS, Hayes SC, Bunting K, Rye AK. Reducing self-stigma in substance abuse through acceptance and commitment therapy: Model, manual development, and pilot outcomes. Addiction research & theory [Internet]. 2008 Apr [cited 2023 Mar 17];16(2):149. Available from: https://www.ncbi.nlm.nih.gov/pmc/articles/PMC5064952/. 10.1080/16066350701850295 PMC5064952 27746709

[6] Corrigan PW, Watson AC, Barr L (2006). The self-stigma of mental illness: implications for self-esteem and self-efficacy. J Soc Clin Psychol.

[7] Luoma JB, Nobles RH, Drake CE, Hayes SC, O’Hair A, Fletcher L et al. Self-Stigma in Substance Abuse: Development of a New Measure. J Psychopathol Behav Assess [Internet]. 2013 Jun 6 [cited 2023 Jun 21];35(2):223. Available from: https://pubmed.ncbi.nlm.nih.gov/23772099/. 10.1007/s10862-012-9323-4 PMC3680138 23772099

[8] Luoma JB, Platt MG (2015). Shame, self-criticism, self-stigma, and compassion in Acceptance and Commitment Therapy. Curr Opin Psychol.

[9] Krafft J, Ferrell J, Levin ME, Twohig MP (2018). Psychological inflexibility and stigma: a meta-analytic review. J Contextual Behav Sci.

[10] Lucena-Santos P, Carvalho SA, Oliveira MS, Pinto-Gouveia J. Body-Image Acceptance and Action Questionnaire: Its deleterious influence on binge eating and psychometric validation. Int J Clin Health Psychol [Internet]. 2017 May 1 [cited 2023 Mar 18];17(2):151. Available from: https://www.ncbi.nlm.nih.gov/pmc/articles/PMC6220905/. 10.1016/j.ijchp.2017.03.001 PMC6220905 30487890

[11] Crapanzano KA, Hammarlund R, Ahmad B, Hunsinger N, Kullar R. The association between perceived stigma and substance use disorder treatment outcomes: a review. Subst Abuse Rehabil [Internet]. 2019 Dec [cited 2023 Mar 18];10:1. Available from: https://www.ncbi.nlm.nih.gov/pmc/articles/PMC6311321/. 10.2147/SAR.S183252 PMC6311321 30643480

[12] Hogue A, Becker SJ, Wenzel K, Henderson CE, Bobek M, Levy S et al. Family Involvement in Treatment and Recovery for Substance Use Disorders among Transition-Age Youth: Research Bedrocks and Opportunities. J Subst Abuse Treat [Internet]. 2021 Oct 1 [cited 2023 Jun 21];129:108402. Available from: https://pubmed.ncbi.nlm.nih.gov/34080559/. 10.1016/j.jsat.2021.108402 PMC8380649 34080559

[13] DiClemente CC, Nidecker M, Bellack AS (2008). Motivation and the stages of change among individuals with severe mental illness and substance abuse disorders. J Subst Abuse Treat.

[14] Bilici R, Yazici E, Tufan AE, Mutlu E, Izci F, Uǧurlu GK. Motivation for treatment in patients with substance use disorder: personal volunteering versus legal/familial enforcement. Neuropsychiatr Dis Treat [Internet]. 2014 Aug 30 [cited 2023 Jun 21];10:1599. Available from: /pmc/articles/PMC4155996/. 10.2147/NDT.S66828 PMC4155996 25210453

[15] Kübler U, Structured Clinical Interview for DSM-IV (SCID). Encyclopedia of Behavioral Medicine [Internet]. 2013 [cited 2023 Mar 18];1919–20. Available from: https://link.springer.com/referenceworkentry/10.1007/978-1-4419-1005-9_66.

[16] Granger DA, Johnson SB, Structured Clinical Interview for DSM-IV (SCID). Encyclopedia of Behavioral Medicine [Internet]. 2013 [cited 2023 Jun 21];1919–20. Available from: https://link.springer.com/referenceworkentry/10.1007/978-1-4419-1005-9_66.

[17] Nagy NES, Ella EIA, Shorab EM, Moneam MHEDA, Tohamy AA. Assessment of addiction management program and predictors of relapse among inpatients of the Psychiatric Institute at Ain Shams University Hospital. Middle East Current Psychiatry. 2022;29(1).

[18] GOSSOP M, DARKE S, GRIFFITHS P, HANDO J, POWIS B, HALL W et al. The Severity of Dependence Scale (SDS): psychometric properties of the SDS in English and Australian samples of heroin, cocaine and amphetamine users. Addiction (Abingdon, England) [Internet]. 1995 [cited 2023 Mar 18];90(5):607–14. Available from: https://pubmed.ncbi.nlm.nih.gov/7795497/. 10.1046/j.1360-0443.1995.9056072.x 7795497

[19] Dawe S, Loxton NJ, Hides L, Kavanagh DJ, Mattick RP. Review of Diagnostic Screening Instruments for Alcohol and Other Drug Use and Other Psychiatric Disorders. 2002 [cited 2023 Mar 18]; Available from: http://www.nada.org.au/media/14712/screening_assessment_review.pdf.

[20] Ouanouche EH, Elmostafi H, Amarat N, Wafaa O, Ryad T, El Hessni A et al. Cannabis and schizophrenia: characterisation of a risk factor in a sample of Moroccan patients hospitalised for psychosis. Middle East Current Psychiatry [Internet]. 2022 Dec 1 [cited 2023 Mar 18];29(1):1–8. Available from: https://mecp.springeropen.com/articles/10.1186/s43045-022-00173-5.

[21] Luoma JB, O’Hair AK, Kohlenberg BS, Hayes SC, Fletcher L. The Development and Psychometric Properties of a New Measure of Perceived Stigma Toward Substance Users. http://dx.doi.org/103109/10826080902864712 [Internet]. 2009 [cited 2023 Mar 18];45(1–2):47–57. Available from: https://www.tandfonline.com/doi/abs/10.3109/10826080902864712. 10.3109/10826080902864712 PMC5067154 20025438

[22] Beaton DE, Bombardier C, Guillemin F, Ferraz MB. Guidelines for the process of cross-cultural adaptation of self-report measures. Spine (Phila Pa 1976) [Internet]. 2000 Dec 15 [cited 2023 Mar 18];25(24):3186–91. Available from: https://pubmed.ncbi.nlm.nih.gov/11124735/. 10.1097/00007632-200012150-00014 11124735

[23] Polit DF, Beck CT. The content validity index: are you sure you know what’s being reported? Critique and recommendations. Res Nurs Health [Internet]. 2006 Oct [cited 2023 Mar 18];29(5):489–97. Available from: https://pubmed.ncbi.nlm.nih.gov/16977646/. 10.1002/nur.20147 16977646

[24] Burns VF, Walsh CA, Smith J. A Qualitative Exploration of Addiction Disclosure and Stigma among Faculty Members in a Canadian University Context. Int J Environ Res Public Health [Internet]. 2021 Jul 2 [cited 2023 Mar 18];18(14). Available from: /pmc/articles/PMC8306368/. 10.3390/ijerph18147274 PMC8306368 34299723

[25] Sattler S, Escande A, Racine E, Göritz AS. Public Stigma Toward People With Drug Addiction: A Factorial Survey. J Stud Alcohol Drugs [Internet]. 2017 [cited 2023 Mar 18];78(3):415–25. Available from: https://pubmed.ncbi.nlm.nih.gov/28499109/. 10.15288/jsad.2017.78.415 28499109

[26] Hammarlund RA, Crapanzano KA, Luce L, Mulligan LA, Ward KM. Review of the effects of self-stigma and perceived social stigma on the treatment-seeking decisions of individuals with drug- and alcohol-use disorders. Subst Abuse Rehabil [Internet]. 2018 Nov [cited 2023 Mar 18];9:115. Available from: /pmc/articles/PMC6260179/. 10.2147/SAR.S183256 PMC6260179 30538599

[27] Merrill JE, Monti PM. Influencers of the Stigma Complex toward Substance Use and Substance Use Disorders. 2015.

[28] Scioli GA, Carmona-Torres JA, Paniccia A, Battista A, Cavicchia I, Bishar RM (2015). A study on the perception of the stigma related to drug use in a sample of Italians and Belgians. Psychol Soc Educ.

[29] Kulesza M, Larimer ME, Rao D. Substance Use Related Stigma: What we Know and the Way Forward. J Addict Behav Ther Rehabil [Internet]. 2013 [cited 2023 Mar 18];2(2). Available from: /pmc/articles/PMC4228689/. 10.4172/2324-9005.1000106 PMC4228689 25401117

[30] Mackenzie CS, Heath PJ, Vogel DL, Chekay R. Age differences in public stigma, self-stigma, and attitudes toward seeking help: A moderated mediation model. J Clin Psychol [Internet]. 2019 Dec 1 [cited 2023 Mar 18];75(12):2259–72. Available from: https://pubmed.ncbi.nlm.nih.gov/31385298/. 10.1002/jclp.22845 31385298

[31] Sattler S, Zolala F, Baneshi MR, Ghasemi J, Amirzadeh Googhari S (2021). Public Stigma toward Female and male opium and heroin users. An experimental test of Attribution Theory and the Familiarity Hypothesis. Front Public Health.

[32] El Rasheed AH, El Sheikh MM, El Missiry MA, Hatata HA, Ahmed N. Addiction stigma among mental health professionals and medical students in Egypt. Addict Disord Their Treat [Internet]. 2016 [cited 2023 May 26];15(4):165–74. Available from: https://journals.lww.com/addictiondisorders/Fulltext/2016/12000/Addiction_Stigma_Among_Mental_Health_Professionals.3.aspx.

[33] Sorsdahl K, Stein DJ, Myers B. Negative attributions towards people with substance use disorders in South Africa: Variation across substances and by gender. BMC Psychiatry [Internet]. 2012 Aug 7 [cited 2023 May 26];12(1):1–8. Available from: https://bmcpsychiatry.biomedcentral.com/articles/10.1186/1471-244X-12-101. 10.1186/1471-244X-12-101 PMC3480848 22871303

[34] Record-High 50% of Americans. Favor Legalizing Marijuana Use [Internet]. [cited 2023 May 26]. Available from: https://news.gallup.com/poll/150149/Record-High-Americans-Favor-Legalizing-Marijuana.aspx.

[35] Cheng CM, Chang CC, Wang J, Der, Chang KC, Ting SY, Lin CY. Negative Impacts of Self-Stigma on the Quality of Life of Patients in Methadone Maintenance Treatment: The Mediated Roles of Psychological Distress and Social Functioning. Int J Environ Res Public Health [Internet]. 2019 Apr 1 [cited 2023 Jun 25];16(7):1299. Available from: /pmc/articles/PMC6480473/. 10.3390/ijerph16071299 PMC6480473 30978986

[36] Hammarlund RA, Crapanzano KA, Luce L, Mulligan LA, Ward KM. Review of the effects of self-stigma and perceived social stigma on the treatment-seeking decisions of individuals with drug- and alcohol-use disorders. Subst Abuse Rehabil [Internet]. 2018 Nov [cited 2023 Jun 25];9:115. Available from: /pmc/articles/PMC6260179/. 10.2147/SAR.S183256 PMC6260179 30538599

[37] Werner P, Stein-Shvachman I, Heinik J. Perceptions of self-stigma and its correlates among older adults with depression: a preliminary study. Int Psychogeriatr [Internet]. 2009 Dec [cited 2023 Mar 18];21(6):1180–9. Available from: https://pubmed.ncbi.nlm.nih.gov/19586565/. 10.1017/S1041610209990470 19586565

[38] Armstrong J, Loneliness. and Perceived Stigmatization Among Older Adults Enrolled in Opiate Substitution Treatment Programs and the Utilization of Mental Health Services. Antioch University Full-Text Dissertations & Theses [Internet]. 2015 Jan 1 [cited 2023 Mar 18]; Available from: https://aura.antioch.edu/etds/246.

[39] Brohan E, Elgie R, Sartorius N, Thornicroft G. Self-stigma, empowerment and perceived discrimination among people with schizophrenia in 14 European countries: the GAMIAN-Europe study. Schizophr Res [Internet]. 2010 Sep [cited 2023 Mar 18];122(1–3):232–8. Available from: https://pubmed.ncbi.nlm.nih.gov/20347271/. 10.1016/j.schres.2010.02.1065 20347271

[40] Biancarelli DL, Biello KB, Childs E, Drainoni M, Salhaney P, Edeza A (2019). Strategies used by people who inject drugs to avoid stigma in healthcare settings. Drug Alcohol Depend.

[41] Paulozzi LJ, Strickler GK, Kreiner PW, Koris CM (2015). Controlled substance prescribing patterns–prescription behavior Surveillance System, eight States, 2013. MMWR Surveill Summ.

[42] Belenko S, Hiller M, Hamilton L. Treating substance use disorders in the criminal justice system. Curr Psychiatry Rep [Internet]. 2013 Nov 17 [cited 2023 Mar 20];15(11):1–11. Available from: https://link.springer.com/article/10.1007/s11920-013-0414-z. 10.1007/s11920-013-0414-z PMC3859122 24132733

[43] Wogen J, Restrepo MT. Human Rights, Stigma, and Substance Use. Health Hum Rights [Internet]. 2020 Jun 1 [cited 2023 Mar 18];22(1):51. Available from: /pmc/articles/PMC7348456/. PMC7348456 32669788

